# Timing matters: overall treatment time, radiotherapy interruptions, and outcomes in glioblastomas—prognostic significance in different biological sub-groups

**DOI:** 10.1007/s11060-025-05215-6

**Published:** 2025-09-25

**Authors:** Paolo Tini, Flavio Donnini, Francesco Marampon, Pierpaolo Pastina, Giovanni Rubino, Giuseppe Battaglia, Salvatore Chibbaro, Alfonso Cerase, Maria Antonietta Mazzei, Isacco Desideri, Giuseppe Minniti

**Affiliations:** 1 Unit of Radiation Oncology, Department of Medicine, Surgery and Neurosciences, University of Siena, Siena, Italy; 2 Radiation Oncology, Policlinico Umberto I, Department of Radiological, Oncological and Pathological Sciences, ″Sapienza″ University of Rome, Rome, Italy; 3 Neurosurgery, Azienda Ospedaliera Universitaria Senese, Siena, Italy; 4 Unit of Neuroradiology, Azienda Ospedaliera Universitaria Senese, Siena, Italy; 5 Unit of Diagnostic Imaging, Department of Medicine, Surgery and Neurosciences, University of Siena, Siena, Italy; 6 Department of Biomedical, Experimental and Clinical Sciences “Mario Serio”, University of Florence, 50134 Florence, Italy; 7 IRCSS Neuromed, Pozzilli, Italy

**Keywords:** Glioblastoma, Overall treatment time, Radiotherapy interruption, MGMT promoter methylation, EGFR amplification

## Abstract

**Background:**

Glioblastoma (GBM) is the most common and aggressive primary malignant brain tumor in adults, with a median overall survival (OS) rarely exceeding 15 months despite multimodal therapy. While established prognostic factors include age, Karnofsky Performance Status (KPS), and molecular features such as MGMT promoter methylation and IDH mutation status, increasing attention has focused on the role of treatment timing as a potentially modifiable prognostic determinant. In particular, Overall Treatment Time (OTT)—the number of calendar days from the first to the last radiotherapy fraction—may impact survival by enabling tumor repopulation when extended or interrupted.

**Methods:**

We conducted a retrospective monocentric cohort study of 166 consecutive adult patients with histologically confirmed IDH-wild-type glioblastoma treated with standard concurrent chemoradiotherapy and adjuvant temozolomide between January 2016 and January 2024. OTT was defined as the total number of days from radiotherapy start to end, including all interruptions. A cutoff of 48 days was adopted based on prior evidence. Patients were stratified according to OTT, number and cause of radiotherapy interruptions, and molecular status (MGMT promoter methylation and EGFR amplification). The primary endpoints were OS and progression-free survival (PFS), analyzed with Kaplan–Meier and Cox regression models.

**Results:**

Median OTT was 43 days (range: 40–65). Patients with OTT ≤ 48 days had a significantly longer median OS than those with OTT > 48 days (20 vs. 10 months, *p* = 0.003). Multivariable Cox regression confirmed OTT > 48 days as an independent negative prognostic factor (HR = 1.41, *p* = 0.009). Multiple interruptions, regardless of cause, further reduced OS, particularly in patients with MGMT-methylated tumors and low EGFR expression. Clinical interruptions—often due to toxicity—were associated with significantly worse outcomes than single technical interruptions. Notably, the negative impact of prolonged OTT was significantly more pronounced in the MGMT-methylated subgroup (p for interaction = 0.018), suggesting a biologically distinct vulnerability to treatment delays.

**Conclusions:**

This study demonstrates that prolonged OTT and radiotherapy interruptions are independently associated with inferior survival in patients with IDH-wild-type glioblastoma, particularly in biologically favorable subgroups such as MGMT-methylated tumors. These findings underscore the importance of strict adherence to treatment schedules and minimizing avoidable delays. Molecular profiling may aid in identifying patients most vulnerable to the adverse effects of treatment prolongation, supporting a more personalized and time-sensitive approach to GBM management. Further prospective validation is warranted.

## Introduction

Glioblastoma (GBM) is the most aggressive primary brain tumor in adults, characterized by rapid proliferation, diffuse infiltration, and marked inter- and intra-tumoral heterogeneity. Despite advances in multimodal therapy—including maximal safe surgical resection followed by concurrent radiotherapy and temozolomide (RT-TMZ), and adjuvant TMZ according to the Stupp protocol—median overall survival (OS) remains dismal, averaging 14–16 months in contemporary cohorts [1]. Increasing attention has focused on Overall Treatment Time (OTT)—defined as the number of days between the first and last radiotherapy fraction—as a modifiable prognostic factor across several malignancies treated with radiotherapy, including head and neck [2], gynecological [3], lung [4] and prostate [5] cancers. Prolonged OTT may enable tumor cell repopulation and reduce radiosensitivity, undermining treatment efficacy—a concept supported by well-established radiobiological principles, and ultimately reflected in inferior clinical outcomes. In glioblastoma, however, evidence remains conflicting. While some studies suggest that extended OTT negatively impacts survival [6], others report no significant association [7, 8], reflecting ongoing uncertainty and the need for further molecularly informed analyses. These discrepancies may reflect methodological heterogeneity, unmeasured confounding factors, and a lack of biomolecular stratification in prior analyses. Crucially, the interaction between treatment timing and tumor biology has been largely underexplored. Prognosis in GBM is traditionally influenced by patient age, Karnofsky Performance Status (KPS), extent of resection, and molecular features such as MGMT promoter methylation and IDH mutation status [9, 10] MGMT promoter methylation is a well-established predictor of responsiveness to alkylating agents such as TMZ [11], while EGFR amplification has been associated with increased proliferative activity and therapeutic resistance [12, 13]. Whether these molecular markers also influence the tumor’s vulnerability to prolonged or interrupted radiotherapy remains unknown.

In this context, we conducted a retrospective cohort study to evaluate the prognostic significance of OTT and radiotherapy interruptions in patients with IDH-wild-type GBM treated with the Stupp protocol. We also investigated whether MGMT methylation and EGFR amplification modulate the impact of treatment timing on OS. By integrating clinical, temporal, and molecular parameters, this study aims to clarify unresolved questions and support evidence-based, individualized treatment scheduling in GBM.

## Materials and methods

### Study design and patient selection

This retrospective, single-center observational study was conducted at the Department of Radiation Oncology, Azienda Ospedaliero-Universitaria Senese (University Hospital of Siena, Italy). We reviewed records of consecutive adult patients with histologically confirmed IDH-wildtype glioblastoma (per 2021 WHO classification [10]) treated between January 2016 and January 2024. Inclusion criteria were: (1) newly diagnosed, histologically confirmed IDH-wildtype GBM; (2) completion of standard concurrent RT-TMZ chemoradiotherapy according the Stupp protocol [1]. Radiotherapy consisted of 60 Gy in 30 fractions (2 Gy per fraction delivered Monday–Friday over ~ 6 weeks) using intensity-modulated techniques. Concurrent temozolomide (TMZ) chemotherapy was administered at 75 mg/m^2^ daily throughout radiotherapy. After completing RT-TMZ, patients received up to 6–12 cycles of adjuvant TMZ (150–200 mg/m^2^ given on 5 consecutive days every 28 days), as per the Stupp protocol [1].; and (3) availability of complete clinical data, including radiotherapy dates, follow- up, and molecular marker status (MGMT promoter methylation and EGFR amplification). Patients were excluded if they received non-standard or palliative-intent treatment, experienced early tumor progression before radiotherapy initiation, or had incomplete data on treatment duration or molecular status.

### Definition of overall treatment time (OTT) and interruptions

Overall Treatment Time was defined as the number of calendar days from the first to the last radiotherapy fraction, inclusive of weekends and any breaks. Because radiotherapy was delivered on weekdays only, the day of week on which treatment started influenced the calendar OTT even in the absence of interruptions. An uninterrupted 30-fraction course lasts ~ 40 days if initiated on a Monday, and ~ 42 days if initiated later in the week (due to intervening weekends). Thus, some variability in OTT (approximately 40–42 days) reflects scheduling logistics rather than true treatment delays.We performed an exploratory analysis comparing patients with 0, 1, or 2 days of unplanned interruption (excluding weekends/holidays). There was no significant OS difference between 0 and 1 day, and 2 days interruptions. Consequently, “Significant” treatment interruptions were defined as any unplanned radiotherapy gap > 2 consecutive weekdays (excluding scheduled off-treatment days like weekends). All interruptions were recorded and classified by number (none, single, or multiple) and cause: clinical interruptions (due to patient-related issues such as hematologic or neurologic toxicity, intercurrent illness, or decline in performance status requiring a pause) versus technical interruptions (due to non-medical factors such as linear accelerator downtime or scheduling/logistical issues).

### Molecular analysis

Tumor molecular profiling was performed on formalin-fixed, paraffin-embedded surgical or biopsy tissue. MGMT promoter methylation status was determined by methylation-specific.

PCR, and EGFR amplification was assessed by fluorescence in situ hybridization (FISH) or next- generation sequencing. Patients were stratified into MGMT-methylated vs. unmethylated and EGFR- amplified vs. non-amplified (normal copy-number, “EGFR-low”) groups for subgroup analyses.

### Outcomes and follow-up

Primary endpoint was overall survival (OS), defined as time from histological diagnosis to death from any cause. Patients were monitored with clinical examinations and MRI every 2–3 months per institutional protocol.

### Statistical analysis

Baseline demographic, clinical, and molecular characteristics were summarized with descriptive statistics. Continuous variables are reported as mean, median, and range; categorical variables as counts and percentages. Survival analyses were performed using the Kaplan–Meier method; differences between groups were assessed by log-rank test. An optimal prognostic cut-off for OTT was identified using maximally selected log-rank statistics (Maximally Selected Rank test), which evaluates all possible cut-off values along the OTT distribution and selects the threshold that maximizes the log-rank chi-square statistic. A Lausen–Schumacher adjustment was applied to correct for multiple testing and avoid over-fitting. A multivariable Cox proportional hazards model was constructed to identify independent predictors of OS. Candidate covariates included OTT (dichotomized at the prognostic cut-off), number of interruptions (≥ 2 vs. <2), interruption cause, MGMT status, EGFR status, age at diagnosis, KPS, and extent of resection (gross total vs. subtotal vs. biopsy). Interaction terms were tested to evaluate whether the prognostic impact of OTT differed by MGMT or EGFR status. Hazard ratios (HR) with 95% confidence intervals (CI) and two-tailed p-values are reported. Statistical analyses were conducted using SPSS v17 and R v4.3.0, with *p* < 0.05 considered significant.

## Results

### Patient characteristics

**Fig. 1:**
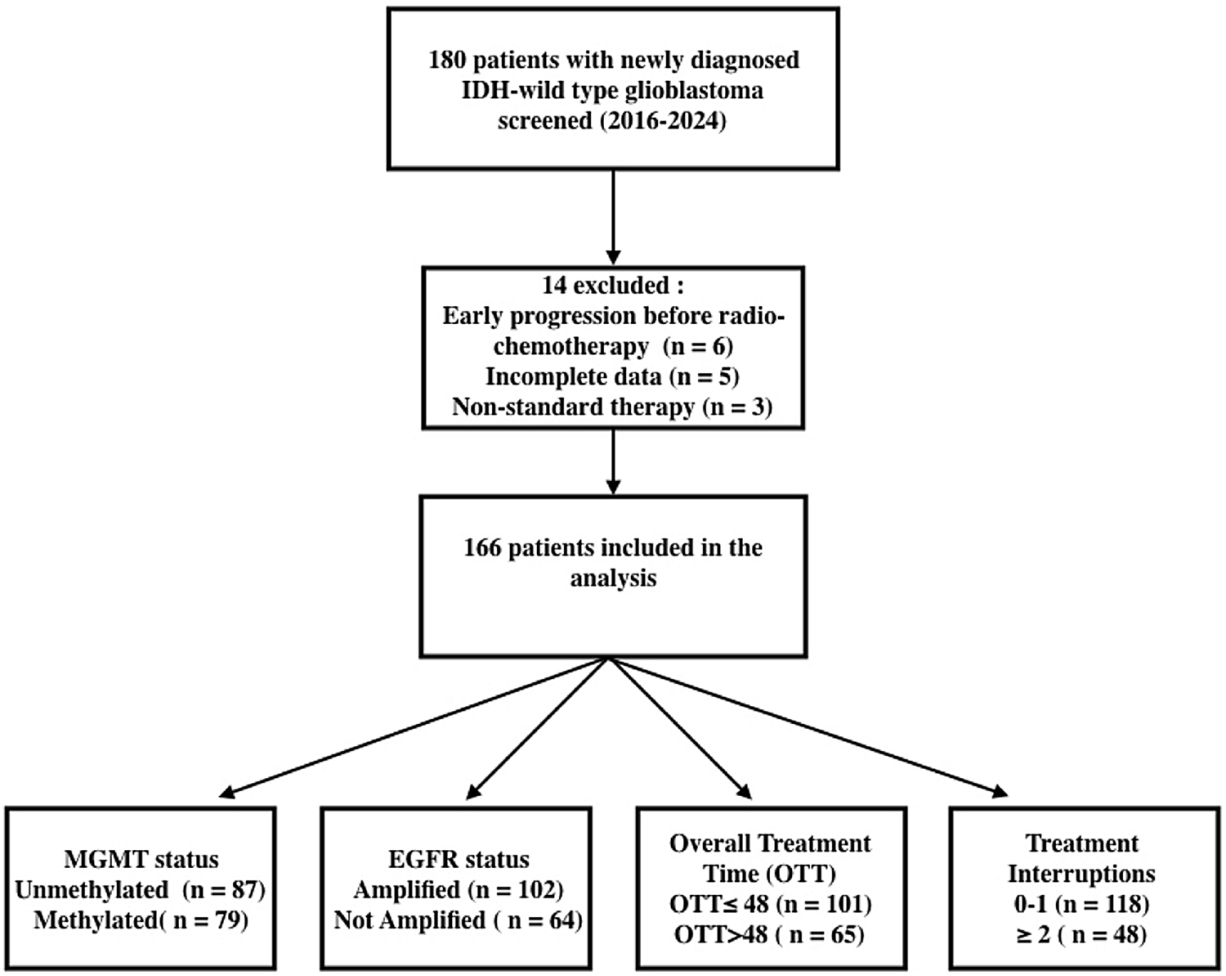
Flow diagram of patient selection. A total of 180 patients with newly diagnosed IDH-wildtype glioblastoma were screened between 2016 and 2024. After exclusions (early progression before chemoradiotherapy, incomplete data, or non-standard therapy), 166 patients met the inclusion criteria and were analyzed”

A total of 166 patients were included in the final analysis. (Fig. 1). The mean age at diagnosis was 63 years (range 26–84), and 106 patients (64%) were male. The median Karnofsky Performance Status at the start of radiotherapy was 80 (range 60–100). In terms of surgery, 50 patients (30%) underwent gross total resection, 80 (48%) had subtotal resection, and 36 (22%) had biopsy only. The median surgery-to-RT start interval was 41 days (range 31–57 days). Key baseline clinical and molecular characteristics are summarized in Table 1.

**Table 1:** Baseline patient and tumor characteristics (*n* = 166), overall and stratified by OTT ≤ 48 days vs. >48 days

Characteristic	Value (*n*, % or range)	OTT ≤ 48 days	OTT > 48 days
Age (years)	63 ys mean (range 26–84)	62 ys	61 ys
Sex	Male 106 (64%); Female 60 (36%)	Male 63; Female 38	Male 43; Female 22
KPS (at radiotherapy start)	80 median (range 60–100)	80 median	80 median
Extent of resection	Gross total 50 (30%); Subtotal 80 (48%); Biopsy 36 (22%)	Gross total 28; Subtotal 50; Biopsy 23;	Gross total 22; Subtotal 30; Biopsy 13;
Surgery-to-RT start interval	Median 41 days (range 31–56 days)	Median 41 days	Median 42 days
MGMT promoter methylation	Methylated 79 (47.6%); Unmethylated 87 (52.4%);	Methylated 49; Unmethylated 52;	Methylated 30; Unmethylated 35;
EGFR amplification	Amplified 102 (60.2%); Not amplified 64 (39.8%);	Amplified 61 Not amplified 40;	Amplified 41 Not amplified 24;

No significant differences were found, *p* > 0.05 for all characteristics

### Molecular profile

MGMT-promoter methylation was detected in 79/166 assessable tumours (47.6%), whereas 87 (52.4%) were unmethylated. EGFR was amplified in 100/166 cases (60.2%); the remaining 66 (39.8%) displayed low/normal copy number.

### Overall treatment time (OTT)

**Fig. 2:**
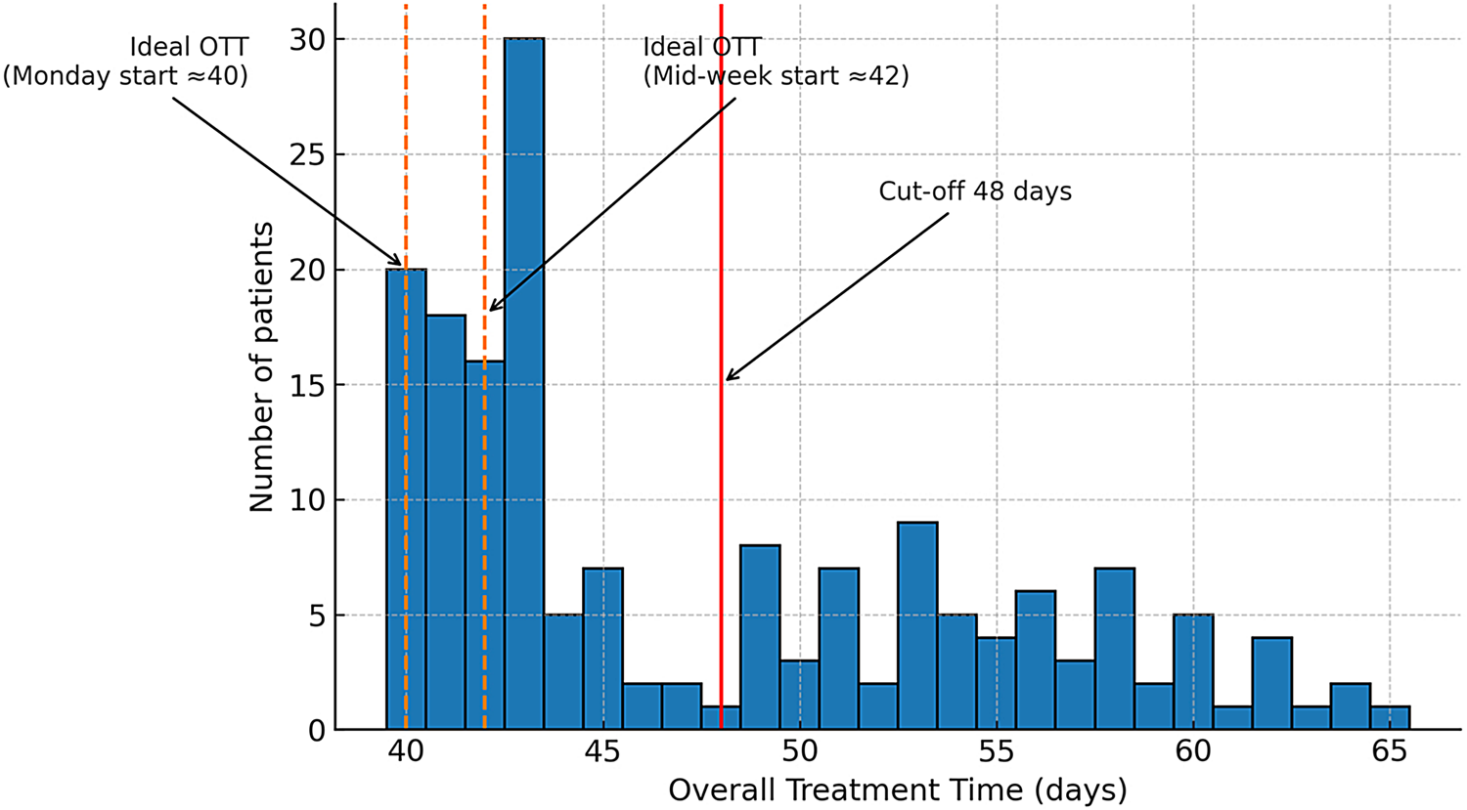
Distribution of Overall Treatment Time (OTT) in the study cohort (n = 166). The histogram shows the number of patients by OTT in days, from the first to the last radiotherapy fraction (including any breaks). Dashed orange lines indicate the idealized calendar OTT for a 30-fraction schedule without interruptions, starting on a Monday (~ 40 days) or mid-week (~ 42 days). The solid red line marks the study-derived prognostic cut-off of 48 days. Most patients without significant treatment breaks completed radiotherapy in 40–45 days, while a subset experienced substantial prolongation of OTT, often due to unplanned interruptions

**Fig. 3:**
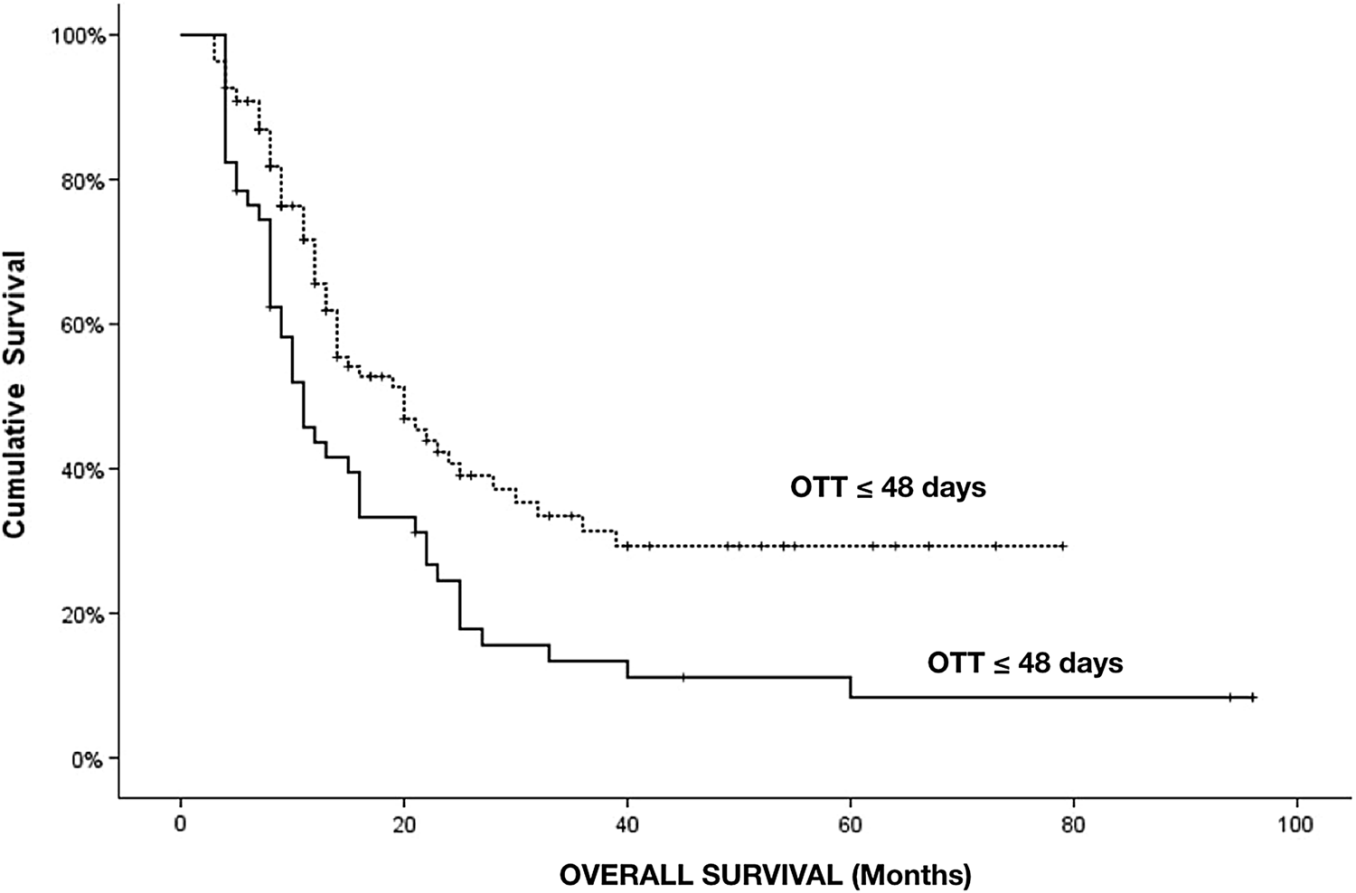
Kaplan–Meier overall survival curves stratified by Overall Treatment Time (OTT ≤ 48 days vs. >48 days). Patients with prolonged OTT beyond 48 days had significantly worse survival, with median OS of 10 months compared to 20 months for those treated within 48 days (p = 0.003)

The median overall treatment time from the first to last radiotherapy fraction (including any breaks) was 43 days (range 40–65 days). Figure 2 illustrates the distribution of OTT in our cohort: most patients without significant breaks finished in about 40–45 days, while a subset of patients had OTT prolonged well beyond 48 days due to interruptions.An optimal prognostic cut-off for OTT was identified using maximally selected log-rank statistics (Maximally Selected Rank test), which evaluates all possible cut-off values along the OTT distribution and selects the threshold that maximizes the log-rank chi-square statistic; a Lausen–Schumacher adjustment was applied to correct for multiple testing and avoid over-fitting. This procedure identified 48 days as the optimal threshold for prognostication. Patients who completed radiotherapy within 48 days had a significantly longer median OS of 20 months compared to 10 months in those exceeding 48 days (log-rank *p* = 0.003) (Fig. 3). The median surgery-to-RT start interval was similar between patients with OTT ≤ 48 days and those with OTT > 48 days (approximately 32 vs. 33 days, *p* = 0.64), indicating no significant delay in treatment initiation for the prolonged OTT group. There were no significant differences between groups in KPS, MGMT status, EGFR status, age, or extent of resection (Table 1).

### Interaction of OTT with molecular biomarkers

**Fig. 4:**
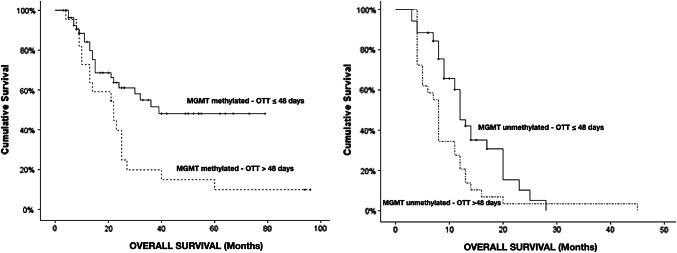
Kaplan–Meier overall survival curves illustrating the impact of OTT stratified by MGMT promoter methylation status. Left: MGMT-methylated tumors—extended OTT > 48 days (n = 30) was associated with markedly worse survival (median OS = 20 months) compared to timely treatment ≤ 48 days (n = 49; median OS 36 months) (log-rank p = 0.005) Right: MGMT-unmethylated tumors (n = 87)—no significant survival difference was seen between OTT > 48 days (n = 35) and ≤ 48 days (n = 52) in this subgroup (log-rank p > 0.05)

The detrimental effect of prolonged treatment was not uniform across molecular subgroups. Notably, in MGMT-methylated tumors, OTT > 48 days was associated with a sharp decrease in survival: median OS fell from 36 months (OTT ≤ 48) to 20 months (OTT > 48), (log-rank *p* = 0.005). Similarly, in EGFR-low (non-amplified) tumors, extended OTT approximately halved median OS (about 28 vs. 14 months, *p* = 0.02). By contrast, in MGMT- unmethylated or EGFR-amplified tumors—biologically more treatment-resistant—prolonged OTT had a lesser and statistically non-significant impact on survival (no significant OS difference between short and long OTT in these subgroups) (Fig. 4). These results suggest that treatment timing is most critical in molecularly favorable GBM, whereas aggressive phenotypes (MGMT-unmethylated or EGFR-amplified) may derive relatively limited benefit from strictly time-adherent therapy.

### Treatment interruptions

A total of 63 significant radiotherapy interruptions were recorded in 57 patients (34% of the cohort). Of these breaks, 40 were due to clinical causes (treatment-related toxicity or patient condition) and 23 were due to technical causes (machine downtime or scheduling issues).

The proportion of patients who temporarily interrupted or discontinued concurrent TMZ during radiotherapy was similar in both OTT groups (*p* = 0.77). Most patients (95% overall) completed the prescribed concurrent TMZ course. Thus, OTT prolongation was not associated with a higher rate of TMZ discontinuation.

### Number of interruptions

Patients experiencing multiple interruptions (≥ 2 treatment breaks) had substantially worse outcomes than those with 0–1 interruptions. The median OS for patients with ≥ 2 significant breaks was only 11 months, compared to 18 months in those with at most one interruption (*p* = 0.004). Subgroup analyses suggested that the impact of interruptions was greatest in the biomarker-favorable groups. For example, MGMT-methylated patients with ≥ 2 interruptions had a median OS of 20 months versus 33 months with 0–1 interruptions, whereas MGMT-unmethylated patients had 10 vs. 13 months, respectively. A similar pattern was observed with EGFR: in EGFR-normal (non amplified) tumors, ≥ 2 interruptions reduced median OS to 14 months (vs. 28 months with none/one break), whereas in EGFR- amplified tumors the difference was smaller (12 vs. 17 months) and not statistically significant. These results are summarized in Table 2.

**Table 2:** Median overall survival (OS) by interruption status, overall and stratified by molecular subgroups

Subgroup	0–1 interruption (median OS, months)	≥ 2 interruptions (median OS, months)	*p*-value
All patients	18 months	11 months	0.004
MGMT-methylated	33 months	20 months	0.031
MGMT-unmethylated	13 months	10 months	0.075
EGFR-low*	28 months	14 months	0.018
EGFR-amplified	17 months	12 months	0.061

EGFR-low = non-amplified (normal EGFR copy number)

### Cause of interruption

When examining interruption cause, we found that even a single clinical interruption (e.g. a pause for toxicity or medical issues) had a noticeable adverse effect on survival, whereas an isolated technical interruption did not. Specifically, compared to patients with no interruptions (median OS = 23 months), those with one clinical interruption had a shorter median OS of 17 months (*p* = 0.041), while those with one technical interruption had a median OS of 20 months (*p* = 0.213, not significant). Patients who experienced multiple interruptions of any cause fared worst, with median OS around 12 months (*p* < 0.01 vs. no interruptions). In summary, a single treatment break due to patient-related factors was associated with significantly reduced survival, whereas equipment-related delays of one instance were less consequential unless multiple such delays occurred.

### Timing of interruptions

We further explored whether the timing of an interruption during the radiotherapy course influenced outcomes, as well as the effect of interruption duration. Among patients who had significant interruptions (*n* = 57), 22 patients experienced their first break in the early phase of RT (weeks 1–3) and the other 35 in the later phase (weeks 4–6). There was no significant difference in OS based on whether interruptions occurred in the early vs. late part of treatment (log-rank *p* = 0.78; i.e., both early and late interruptions were associated with similar survival detriments).

### Multivariable analysis

On Cox proportional-hazards modeling, five factors emerged as independent prognostic predictors for OS (Table 3). MGMT promoter methylation was associated with improved survival (HR 0.55, 95% CI 0.40–0.75, *p* < 0.001), reflecting the known survival benefit in MGMT-methylated GBMs. Older age predicted worse survival (HR 1.03 per year, CI 1.01–1.05, *p* = 0.021). KPS was a strong predictor: for KPS 100 − 80%, the hazard of death decreased (HR ~ 0.89 per 10-point, CI 0.85–0.93, *p* < 0.001), underscoring the protective effect of higher performance status. Importantly, treatment timing factors, except for interval of time to start of treatment, remained significant in the multivariate model: OTT > 48 days independently conferred a higher risk of death (HR 1.41, CI 1.09–1.82, *p* = 0.009), as did having ≥ 2 interruptions (HR 1.61, CI 1.18–2.34, *p* = 0.004), after adjusting for all other variables. We tested for interactions and found a significant interaction between OTT and MGMT status (*p* = 0.018)—indicating that the harm of prolonged OTT was significantly more pronounced in MGMT-methylated tumors than in unmethylated ones, consistent with the subgroup findings above. Conversely, the OTT × EGFR interaction term was not significant (*p* > 0.1), suggesting no strong effect modification by EGFR status. We also note that the interval from surgery to radiotherapy start was not a significant predictor of OS in this multivariable analysis (*p* = 0.45), implying that moderate variations in time to treatment initiation did not independently influence survival outcomes in our cohort. In an alternative Cox model incorporating interruption cause (instead of simply number), we observed that clinical interruptions were the critical driver: a single clinical interruption was associated with a 39% increase in hazard (HR ~ 1.39, CI ~ 1.01–1.91, *p* = 0.042), whereas single technical interruptions did not significantly affect OS. Multiple technical interruptions appeared to adversely affect survival only within the EGFR-low subgroup and did not reach independent significance in the overall model.

**Table 3:** Multivariable Cox regression analysis for overall survival (OS)

Variable	HR	95% CI	*p*-value
MGMT promoter methylated	0.55	0.40–0.75	< 0.001
Age at diagnosis (per year)	1.03	1.01–1.05	0.021
KPS (per 10-point increase)	0.89	0.85–0.93	< 0.001
OTT > 48 days	1.41	1.09–1.82	0.009
≥ 2 interruptions	1.61	1.18–2.34	0.004
Time from surgery to RT start date	0.95	0.63–1.35	n.s.

*HR* hazard ratio, *CI* confidence interval, *KPS* Karnofsky Performance Status, *OTT* Overall Treatment Time

## Discussion

The present study expands on the seminal observation that treatment timing influences outcomes in glioblastoma (GBM) [6, 7, 14] by demonstrating that an Overall Treatment Time (OTT) > 48 days—equivalent to extending the standard six-week chemoradiation course by more than one week—independently predicts inferior overall survival (OS). Importantly, our data show that the detrimental effect of protracted treatment is not uniform across the molecular spectrum of GBM but is magnified in tumours with MGMT-promoter methylation and low/normal EGFR copy number. These findings add a biological dimension to the long-standing clinical imperative of maintaining schedule integrity in radiotherapy. From a radiobiological perspective, even a relatively modest prolongation of approximately six days can have a substantial impact. This interval allows additional opportunities for accelerated repopulation of clonogenic tumor cells between fractions, a process well documented in rapidly proliferating malignancies such as GBM [15]. Given the high intrinsic proliferation rate and short potential doubling time of GBM, such delays may erode the therapeutic gains achieved by concurrent chemoradiation. Furthermore, a prolonged OTT may serve as a surrogate for other adverse factors, including treatment-related toxicity, intercurrent illness, or overall frailty—conditions that not only cause interruptions but may also directly compromise patient resilience and survival. Our multivariable analysis adjusted for major known prognostic factors, including performance status, supporting an independent effect of OTT; however, the potential contribution of unmeasured clinical covariates cannot be excluded. Our results suggest that MGMT-methylated patients—intrinsically more sensitive to temozolomide due to epigenetic silencing of the MGMT DNA-repair enzyme, and, to some extent, radiation—are particularly vulnerable to this time-dependent loss of tumor control. The survival penalty associated with OTT > 48 days was on the order of 12–17 months in the MGMT-methylated subgroup, a striking finding that implies these tumors rapidly lose ground when treatment is protracted. Conversely, MGMT-unmethylated and EGFR-amplified tumors—often characterized by radio- and chemoresistant phenotypes driven by enhanced DNA damage response and pro-survival signaling—showed a much less pronounced and non-significant decrement in survival with OTT prolongation. Collectively, these patterns suggest that biologically favorable GBM subtypes derive maximal benefit from an uninterrupted, intensive treatment schedule, whereas intrinsically resistant tumors may be relatively indifferent to modest delays (because their outcomes are poor regardless of slight timing differences). Our analysis of treatment interruptions further refines this picture of timing and tumor biology. Even a single unplanned break due to clinical reasons (such as neurotoxicity) carried a significant survival cost, which likely reflects both the detrimental effect of treatment discontinuity and the fact that such interruptions often signal a more fragile patient or more aggressive disease course [16]. Clinical interruptions may therefore represent a composite marker of treatment tolerance and evolving performance status during the radio-chemotherapy course. On the other hand, an isolated technical interruption (e.g., one machine maintenance delay) had a negligible impact on OS in our cohort, suggesting that the schedule can absorb a one-time technical delay if treatment is otherwise delivered on time. However, when multiple technical failures accumulated (e.g., repeated machine downtimes), we observed a survival decline in the EGFR-low and MGMT-methylated subgroups and a trend toward worse outcomes overall. This underscores two practical points for clinical practice:

(1) The importance of robust supportive care and toxicity management to prevent or minimize patient-related breaks. When clinically feasible and safe, hospitalized patients may benefit from continuing RT to avoid OTT prolongation, provided that infection control and patient stability are ensured. (2) The need for reliable radiotherapy infrastructure and contingency plans (backup machines, make-up sessions on weekends, etc.) to quickly compensate for any technical downtime.

In summary, not all interruptions are equal—patient-related interruptions are particularly harmful and should be avoided through proactive interventions (antiemetics, hematopoietic growth factors, etc.), whereas the system should be optimized to minimize technical delays and to make up missed sessions promptly when they occur.

Our results align with prior multicentre series reporting a 7–10% reduction in OS for each week of OTT prolongation, yet they add granularity by integrating molecular data [17]. Few previous studies have analysed the interaction between MGMT status and treatment timing; the significant interaction term in our multivariable model supports the concept that schedule adherence is an essential component of “precision timing” for chemosensitive tumours.

## Strengths and limitations

Strengths of the study include a homogeneous treatment protocol, comprehensive recording of interruption cause, and incorporation of two clinically actionable biomarkers. Nonetheless, several limitations must temper interpretation. The retrospective, single-centre design introduces inevitable selection and information biases; The higher proportion of MGMT-methylated tumors in our cohort (47%) compared with other data reported, for example, could represent biases in patient selection for standard chemoradiotherapy. Unmethylated patients generally are more prone to early progression and consequently were more often excluded from chemo-radiotherapy. Our cut-off was identified using maximally selected log-rank statistics and ideally should be validated in an independent dataset. We also tested the previously reported 46-day threshold from literature [7], which yielded consistent but slightly less pronounced results. Although the sample size (*n* = 166) is among the largest single-institution series focused on OTT, subgroup analyses—particularly those stratified by both biomarkers and interruption type—remain underpowered. External validation in larger, multicentre cohorts is warranted.

## Conclusion

This study confirms OTT and interruption burden as modifiable, independent determinants of survival in GBM and reveals that their impact is conditioned by molecular phenotype. Maintaining an OTT ≤ 48 days and preventing multiple or clinically driven pauses should be prioritised, particularly in MGMT-methylated and EGFR-low tumours where the absolute survival benefit is greatest. From an organisational standpoint, our data argue for embedding OTT metrics into quality-assurance dashboards and for real-time monitoring systems that flag impending threshold breaches. Prophylactic measures might include intensified haematologic surveillance for MGMT-methylated patients, early initiation of growth-factor support, and contingency scheduling (accelerated make-up fractions, hypofractionated boosts, or weekend treatments to counteract unavoidable gaps) to accommodate unforeseen machine downtime. These findings support integrating schedule fidelity into precision-oncology paradigms and lay the groundwork for molecularly stratified trials aimed at mitigating time-related therapeutic attrition.

## Data Availability

No datasets were generated or analysed during the current study.
